# Effects of Polyoxymethylene Fiber on Mechanical Properties of Seawater Sea-Sand Concrete with Different Ages

**DOI:** 10.3390/polym14173472

**Published:** 2022-08-25

**Authors:** Fei Wang, Jianmin Hua, Xuanyi Xue, Neng Wang, Yunhang Yao

**Affiliations:** 1 School of Civil Engineering, Chongqing University, Chongqing 400045, China; 2 Key Laboratory of New Technology for Construction of Cities in Mountain Area, Ministry of Education, Chongqing University, Chongqing 400045, China; 3 School of Management Science and Real Estate, Chongqing University, Chongqing 400045, China

**Keywords:** polyoxymethylene (POM) fiber, seawater sea–sand concrete, concrete age, workability, mechanical property

## Abstract

Workability and mechanical properties of the seawater sea–sand concrete (SWSSC) were similar to those of ordinary concrete made with freshwater and river sand, which had a wide application in structures. Since the polyoxymethylene (POM) fiber performed the outstanding alkali resistance and durability, POM fibers were added in SWSSC in this study to enhance the mechanical properties. Moreover, the mechanical properties of concrete during the early age have significant effects on the construction phase. The experiment, including 96 test specimens, was conducted to clarify effects of POM fibers on mechanical properties of SWSSC with different ages. The cube compressive, axial compressive, splitting tensile, and flexural tests of NF and POM0.6 SWSSC were conducted. Based on test results, the predictive equations were proposed to quantify relations between concrete age and mechanical properties of NF and POM0.6 SWSSC. Effects of the concrete age on ratios *f_tT_*/*f_cT_* and *f_fT_*/*f_cT_* were investigated and quantified through proposed equations. Failure performances of NF and POM0.6 SWSSC specimens with different ages were analyzed. The microstructure of POM0.6 SWSSC specimens was observed, and the reinforcing mechanism of POM fibers was further explained.

## 1. Introduction

For concrete widely used in engineering structures, hydration of cement creates an alkali environment, where the passivation layer is built on the surface of carbon steel reinforcement. The passivation layer can protect the carbon steel reinforcement from corrosion effectively, which improves the durability of reinforced concrete (RC) structures. According to the experimental investigations conducted by many researchers [1,2,3,4], the workability and mechanical properties of the seawater sea–sand concrete (SWSSC) were similar to those of ordinary concrete made with freshwater and river sand. Therefore, it is possible to use SWSSC in engineering widely. It is noteworthy that the contents of chloride ion in untreated seawater and sea–sand are relatively high. The passivation layer of steel reinforcement could be penetrated by the chloride ions, which reduces the aforementioned protection effect directly. For RC structures consisting of SWSSC and carbon steel reinforcement, the durability could be reduced significantly by the reinforcement corrosion due to the chloride ion in untreated seawater and sea–sand. To strengthen the service performance of RC structures, many reinforcements material with outstanding corrosion resistance were proposed with advancement in processing technology, such as fiber-reinforced polymer bars (FRPBs) [5,6], steel-FRP composite bars (SFCBs) [7], epoxy-coated bars (ECBs) [8], bimetallic steel [9,10], and stainless-clad bimetallic steel bars (SCBSBs) [11,12,13,14,15,16,17,18]. The application of steel with the outstanding corrosion resistance could relieve the above corrosion issue caused by chloride ion in untreated seawater and sea–sand. When the corrosion issue was solved properly, SWSSC would have a wide application in RC structures [19]. With the application of untreated seawater and sea–sand, the consumption of freshwater and river sand could be reduced, correspondingly. Then, the environmental problems caused by the over exploitation of freshwater and river sand will be solved from the root. Furthermore, the tropic islands or reefs could be far away from lands [20]. Therefore, A large amount of freshwater and river sand required for construction would be transported to the construction site at a high transportation cost [21,22]. The local materials (seawater and sea–sand) were encouraged [23]. Considering the potential for engineering application, the SWSSC became one of the hotspots of research recently [19]. Limeira et al. [3] investigated the mechanical and durability properties of concretes fabricated with dredged marine sand as a fine granular corrector in partial substitution of raw sand designed for harbor pavements. Li et al. [24] studied the ordinary Portland cement hydration in distilled water and seawater and the corresponding evolution of solid phases. Guo et al. [1] found that the mechanical properties of concrete using untreated sea sand and seawater are basically the same as those of ordinary concrete. Li et al. [25] made seawater sea–sand slag concrete and studied its high temperature resistance. It is worth noting that because of the NaCl and K_2_SO_4_ in the untreated seawater and sea–sand, the strength properties of SWSSC developed faster than those of ordinary concrete in an early age [26,27]. It was meaningful to investigate the mechanical properties of SWSSC with different ages experimentally to guide the design and construction of SWSSC structures.

To increase the crackling resistance and mechanical properties, the fiber was widely used in concrete [28,29,30,31]. The alkali environment in concrete requires a good alkali resistance of added fiber. Furthermore, to guarantee the resistance of RC structures during the whole service cycle, fibers must perform outstanding durability. To meet the above requirements, the polyoxymethylene (POM) fiber was considered in this study. The POM fiber is a new kind of polymer fiber with outstanding alkali resistance, which also performs good durability. Furthermore, the effect of chloride ion in untreated seawater and seas–and on the mechanical performance of the POM fiber could be ignored. Given the aforementioned features, the POM fiber has a wide application in SWSSC. The mechanical properties of POM fibers are different from those of steel, basalt, and polypropylene (PP) fibers, which might lead to differences in the effects of fibers on the performance of SWSSC. Based on the authors’ knowledge, the research on SWSSC reinforced with POM fibers was relatively rare. The fibers used in SWSSC led to changes in the mixture, which might affect the early-age mechanical performance of SWSSC. Considering that the mechanical performance of SWSSC at an early age is different from that of ordinary concrete, the SWSSC reinforced with POM fibers might have a more complex early-age performance. The mechanical performance of concrete at early age has a significant effect on the construction progress. Above all, in order to promote the engineering application of SWSSC with POM fibers, it is meant to clarify the effects of POM fibers on the mechanical performance of SWSSC at an early age. 

In conclusion, POM fiber can affect the mechanical properties of SWSSC at an early age, and it is particularly important to clarify the early-age properties of POM-SWSSC for construction design. However, the research on this issue was limited. In order to clarify the influence mechanism of POM fiber on the early mechanical properties of SWSSC, the workability and mechanical properties of SWSSC with and without POM fibers were investigated experimentally in this study. Details of seawater, sea–sand, and POM fibers were introduced in Section 2. The effect of POM fibers on the workability of SWSSC was studied experimentally in Section 3. Given the references in the code GB/T 50081-2002 [32], the cube compressive, axial compressive, splitting tensile, and flexural tests of SWSSC with and without POM fibers were conducted. Based on test results, the effects of POM fibers on mechanical properties, strain–stress relationship, and failure performances of SWSSC are discussed in Section 4, Section 5 and Section 6, and the reinforcing mechanism of POM fibers was explained from the microstructure. 

## 2. Property of Material

### 2.1. POM Fiber

**Figure 1:**
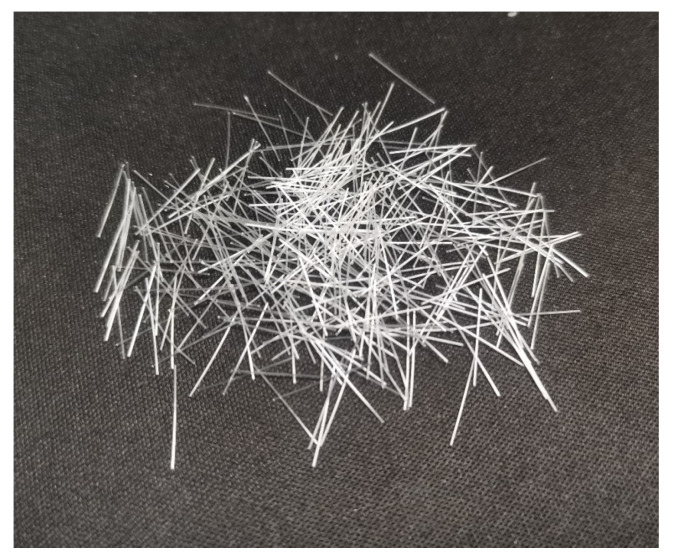
POM fiber.

To improve the mechanical performance of SWSSC, the POM fiber was selected in this study. The POM fiber was produced by the Yuntianhua Co., Ltd., Yunnan, China, and the details are introduced in Table 1. The heat deflection temperature of the POM fiber was 95 °C. As shown in Figure 1, the diameter and length of the POM fiber were 0.2 and 12 mm, respectively. At present, many kinds of fibers have been used to improve the performance of concrete. Since the SWSSC contained an amount of chloride ions and has strong alkalinity, the POM fiber with strong corrosion resistance and alkali resistance can better meet the requirements. However, the mechanical properties of POM fibers were not the best compared with other fibers. To clarify the properties of POM fiber objectively, a comparison among different fibers was performed, as shown in Table 1. The PP fiber was the polypropylene fiber introduced in [29]. The tensile strength of POM fibers was larger than that of PP fibers and smaller than that of glass, carbon, and basalt fibers. Except for steel and PP fibers, the POM fiber performed the largest elongation. The melting point of POM fibers was lower than that of other fibers. The elastic modulus and density of POM fibers were larger than those of PP fibers and lower than those of other fibers. As for steel fibers, they performed outstanding mechanical properties. However, considering the corrosion issue, the durability of steel fibers was much lower than that of other fibers. The POM fiber performed outstanding alkali resistance, which promoted its wide application in concrete structures. Generally, the usage of fibers reduced the workability of concrete [33]. The POM fiber used in this study was monofilament, with smooth surface and certain rigidity, which was not easy to agglomerate. Therefore, the POM fiber can reduce the adverse effect of fibers on the workability of concrete. As the properties of POM fibers were different from those of other fibers, it was meaningful to investigate the effects of POM fibers on the mechanical properties of SWSSC with different ages. 

### 2.2. Sea–Sand and Seawater

**Figure 2:**
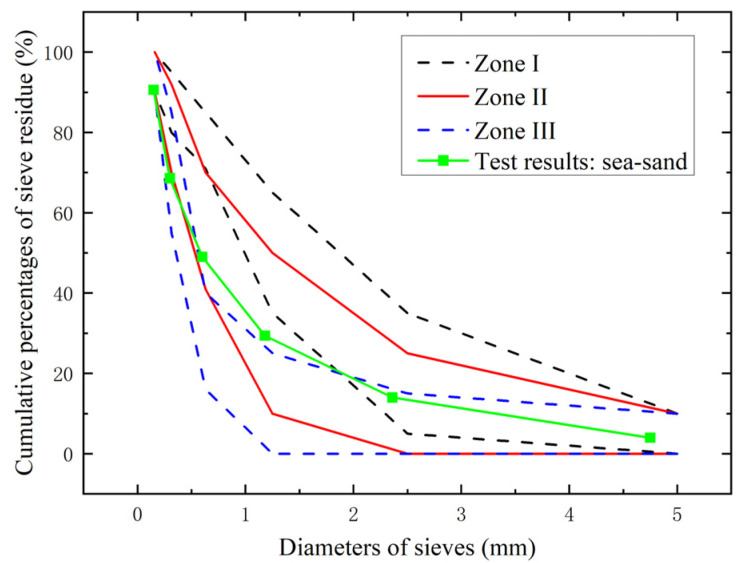
Screening test results of sea–sand.

The seawater and sea–sand could be purified to reduce the content of chloride ions. However, the purification treatment led to large consumption of fresh water, which was against the original intention of the application of seawater and sea–sand. Therefore, the seawater and sea–sand used in this experiment were all untreated. The sea–sand was taken from Guangzhou (113.17° E, 23.79° N) in Guangdong Province, China, and the seawater was taken from Quanzhou (118.36° E, 24.56° N) in Fujian Province, China. Details of the chemical composition of the seawater and sea–sand are introduced in Table 2. It shows that the ion contents of seawater were much higher than those of fresh water. The sand category had a noteworthy effect on the workability and mechanical performance of concrete. To clarify the sand category of the sea–sand used in this experiment, a screening test was conducted to obtain the fineness modulus *M_x_* before the SWSSC production. The *M_x_* was obtained using Equation (1), where *β*_1_, *β*_2_, *β*_3_, *β*_4_, *β*_5_ and *β*_6_ denoted the cumulative percentages of sieve residue for sieves with diameters of 4.75, 2.36, 1.18, 0.6, 0.3, and 0.15 mm, respectively. Results of the screening test are shown in Figure 2, where *M_x_* = 2.41. The Zone I, II, and III were obtained based on JGJ 52-2006 [35]. The cumulative percentage of sieve residue vs. diameters of sieves curve of the sea–sand distributed in Zone II. Hence, the test results stated that the sea–sand met the requirements of the medium sand category. The sea–sand could be used to produce the SWSSC specimens of mechanical tests.

(1) $$\mu_{f}=\frac{\left(\beta_{2}+\beta_{3}+\beta_{4}+\beta_{5}+\beta_{6}\right)-5\beta_{1}}{100-\beta_{1}}$$

### 2.3. Concrete Mixture

To investigate the effects of the POM fiber on mechanical properties of SWSSC with different ages, two different mixtures were considered in this study, as shown in Table 3. NF denoted the SWSSC with no POM fiber, which was the control group. POM0.6 denoted that the volume fraction of POM fibers used in SWSSC was 0.6%. Based on the pre-experiment, the optimal value of volume fraction of POM fibers was about 0.6%. Therefore, the POM0.6 mixture was considered in this experiment to investigate effects of POM fibers on the mechanical performance of SWSSC with different ages. To control the variable, SWSSC were prepared under the same laboratory condition. As for the SWSSC mixing process, the cement, sea–sand, fly ash, mineral powder, and coarse aggregate were dry-mixed for about 3 min. Next, the seawater was added into the mixture. In the end, POM fibers were added into the mixture. Then, workability of the fresh mixture was investigated. Test results are introduced in Section 4. 

## 3. Workability

**Figure 3:**
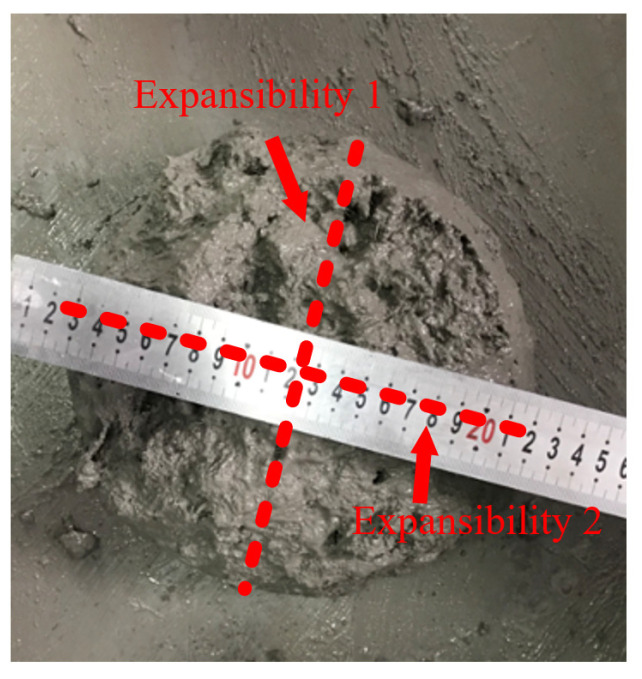
Expansibility test of SWSSC.

The workability of concrete had an important effect on the quality of construction. For the wider application of SWSSC in actual structures, it is necessary to clarify the workability of SWSSC with and without POM fiber. The W/C ratios of the aforementioned NF and POM0.6 SWSSC mixtures were controlled to be the same. The slump and expansibility tests were conducted to quantify the workability of these two different mixtures. For expansibility, the test results of two directions were obtained. The mean value of expansibility 1 and 2 were selected to quantify the expansibility of different mixtures, as shown in Figure 3. Test results of slump and expansibility were obtained, as shown in Table 4, indicating that the application of POM fibers reduced the workability of SWSSC. Compared with those of NF mixture, the slump and expansibility of the POM0.6 mixture decreased by 25.7% and 20.7%, respectively. Compared with the workability of SWSSC with recycled coarse aggregates (SSRCA) introduced in [26], the workability of the NF and POM0.6 SWSSC in this study was better than that of C20 and C30 SSRCA and worse than that of C40 and C50 SSRCA. 

## 4. Mechanical Test Procedure

**Figure 4:**
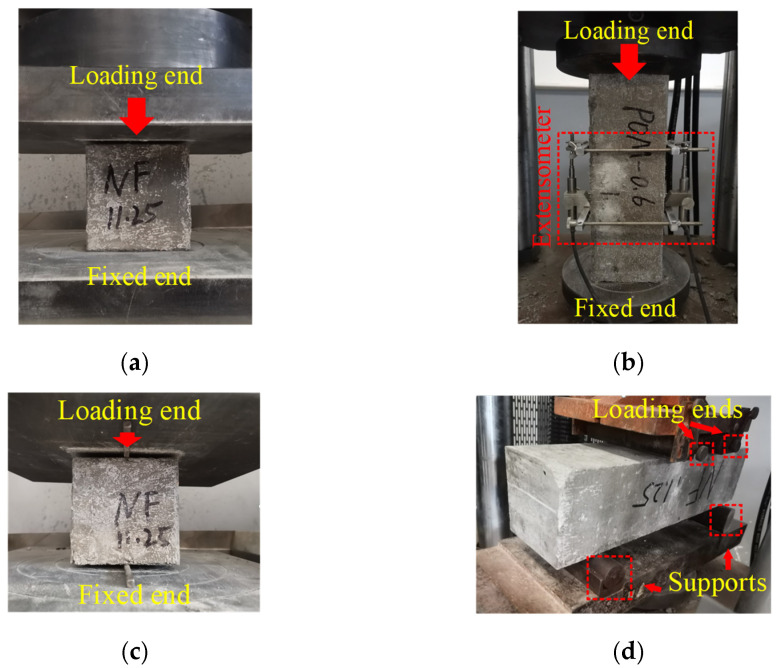
Different mechanical tests: (**a**) cube compression test; (**b**) axial compressive test; (**c**) splitting tensile test; and (**d**) flexural test.

Different concrete tests were included in this study to clarify the influence of ages on the mechanical properties of NF and POM0.6 SWSSC comprehensively. In this study, mechanical properties of NF and POM0.6 SWSSCs with 3, 7, 14, and 28 d were investigated. The cube compressive, axial compressive, splitting tensile, and flexural tests were conducted in accordance to the code GB/T 50081-2002 [32], as shown in Figure 4. For the cube and axial compressive test, the loading rate was set as 0.5 MPa/s. Then, the cube compressive strength *f_cu_* and axial compressive strength *f_c_* were obtained using Equations (2) and (3), where *A* and *F* denoted the bearing area and failure load of test specimens, respectively. For splitting tensile and flexural tests, the loading rate was controlled to be 0.05 MPa/s. Then, the splitting tensile strength *f_s_* and flexural strength *f_f_* were obtained using Equations (4) and (5), where *A_s_*, *b*, and *h* denoted the splitting area of test specimens, height, and width of the cross section, respectively, and *l* denoted the span between supports. Given the references in the code GB/T 50081-2002 [32], the dimensions of test specimens were determined. For cube and axial compressive test specimens, the dimensions were 100 mm × 100 mm × 100 mm and 100 mm × 100 mm × 300 mm, respectively. For splitting tensile test specimens, the dimension was 100 mm × 100 mm × 100 mm. For flexural test specimens, the dimension was 100 mm × 100 mm × 400 mm. The NF and POM0.6 SWSSC mixtures were poured into molds, after which the vibration table was used to compact the SWSSC in molds. After a day, specimens were removed from the molds. All the test specimens were cured in a standard curing room for different ages (3 d, 7 d, 14 d, and 28 d) with 20 ± 2 °C and 95% relative humidity. Next, the mechanical properties of NF and POM0.6 SWSSC specimens were obtained through the mechanical tests. Considering the dimensions of the test specimens used in this study and references in the code GB/T 50081-2002 [32], the conversion factors were selected. For *f_cu_*, *f_c_*, *f_t_*, and *f_f_*, the conversion factors were 0.95, 0.95, 0.85, and 0.85, respectively. For the aforementioned mechanical tests, three specimens were prepared for each kind of concrete mixture and age to reduce the effect of uncertainty of SWSSC on mechanical properties.

(2) $$f_{cu}=\frac{F}{A}$$

(3) $$f_{c}=\frac{F}{A}$$

(4) $$f_{t}=\frac{2F}{\pi A_{s}}=0.637\frac{F}{A_{s}}$$

(5) $$f_{f}=\frac{Fl}{bh^{2}}$$

## 5. Mechanical Test Results

### 5.1. Cube Compressive Property

**Figure 5:**
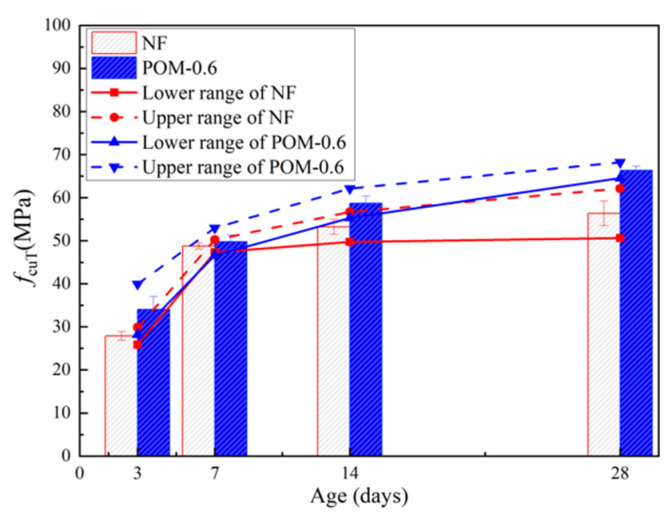
Test results of *f_cuT_* for NF and POM0.6 SWSSC specimens with different concrete ages.

**Figure 6:**
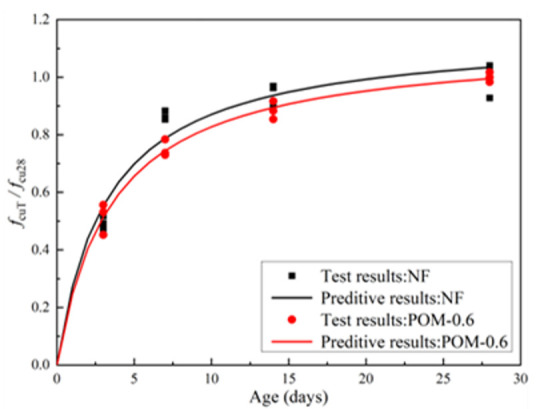
Relations between *f*_cuT_/*f*_cu28_ and concrete age.

After the cube compressive test, test results of NF and POM0.6 SWSSC specimens of different ages were obtained, as shown in Table 5. The specimen’s name NF-3-1 denoted that the concrete mixture was NF, concrete age was 3 d, and the number was 1. For the cube compressive strength (*f_cuT_*) of the test specimens of different ages, the standard deviation varied from 0.72 to 2.93 and the coefficient of variation (COV) ranged from 0.0138 to 0.0859. The relationship between *f_cuT_* and age is shown in Figure 5. The error bar and 95% confidence interval were marked. With increases in concrete age, *f_cuT_* of NF and POM0.6 SWSSC specimens increased, correspondingly. *f_cuT_* increased greatly in the first 7 days and then increased slowly. On the seventh day, the *f_cuT_* of NF and POM0.6 SWSSC increased to 86.5% and 75.0% of the *f_cu_*, respectively. The lower and upper ranges of POM0.6 SWSSC specimens with different ages were larger than those of NF SWSSC specimens with different ages, except for the lower ranges of NF and POM0.6 SWSSC specimens with 7 d. Based on test results of NF and POM0.6 SWSSC specimens of different ages, the POM fiber had an enhancing effect on *f_cuT_*. To eliminate the influence of difference in strength value, the dimensionless processing was performed, as shown in Figure 6, by giving results of *f_cuT_*/*f_cu28_*. It could be concluded that *f_cuT_* of NF SWSSC specimens developed faster than that of POM0.6 SWSSC specimens. To predict the *f_cuT_* of NF and POM0.6 SWSSC accurately, the predictive equations were proposed in this study. Based on the cube compressive test results, Equations (6) and (7) are proposed to quantify *f_cuT_* of NF and POM0.6 SWSSC with different ages, with R^2^ = 0.97 and 0.98, respectively, where *T* denoted age of SWSSC. Comparison between test results and the prediction by Equations (6) and (7) are conducted, as shown in Figure 6. The results indicated that Equations (6) and (7) are used to predict the *f_cu_* of NF and POM0.6 SWSSC with different ages accurately. 

**Figure 7:**
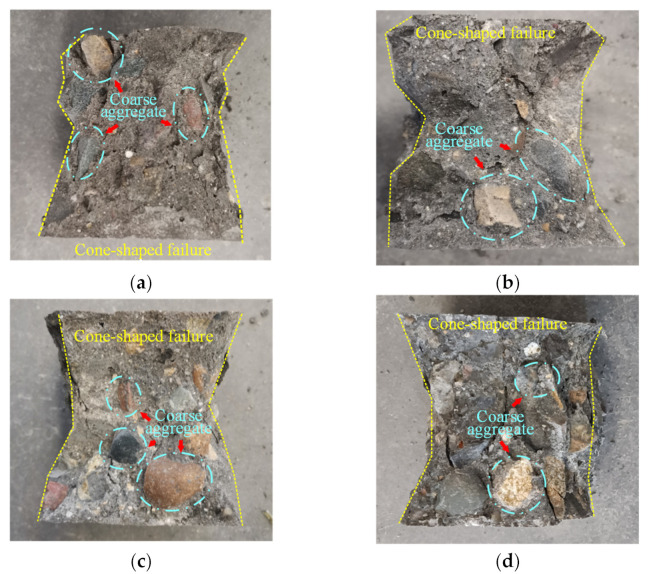
Failure performance of NF specimens with different ages after cube compressive test: (**a**) 3 d; (**b**) 7 d; (**c**) 14 d; and (**d**) 28 d.

**Figure 8:**
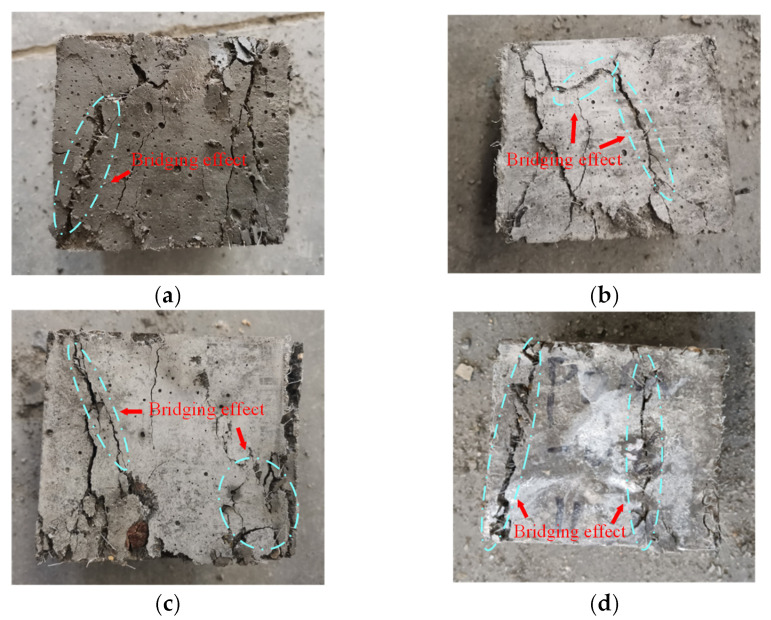
Failure performance of POM0.6 specimens with different ages after cube compressive test: (**a**) 3 d; (**b**) 7 d; (**c**) 14 d; and (**d**) 28 d.

After the cube compressive test, failure performances of NF and POM0.6 SWSSC specimens with different ages were obtained, as shown in Figure 7 and Figure 8. For NF SWSSC specimens with different ages, the typical cone-shaped failure performance was observed. When the concrete age was 3 d, the strength of cement was relatively low and still increased. The crack occurred in the cement and did not pass through the coarse aggregate. After the cube compressive test, the coarse aggregate in test specimen mostly remained intact. With the increase in concrete age, the strength of cement increased correspondingly. For NF SWSSC specimens with 14 and 28 d, cracks in cement passed through the coarse aggregate, as shown in Figure 7. Cracks in the direction of tensile force were observed in POM0.6 SWSSC specimens. The connection constructed by POM fibers restrained the development of cracks. No obvious concrete spalling was observed because of the aforementioned connection. Therefore, after the cube compressive test, the integrity of POM0.6 SWSSC specimens with different ages almost remained, which was different from that of NF SWSSC specimens with different ages.
NF  $f_{\mathrm{cu}T}=\left(\frac{T}{2.822+0.8662T}\right)f_{\mathrm{cu}28}$
  R^2^ = 0.98  (6)POM0.6  $f_{\mathrm{cu}T}=\left(\frac{T}{3.16+0.8922T}\right)f_{\mathrm{cu}28}$
  R^2^ = 0.99  (7)

### 5.2. Axial Compressive Property

**Figure 9:**
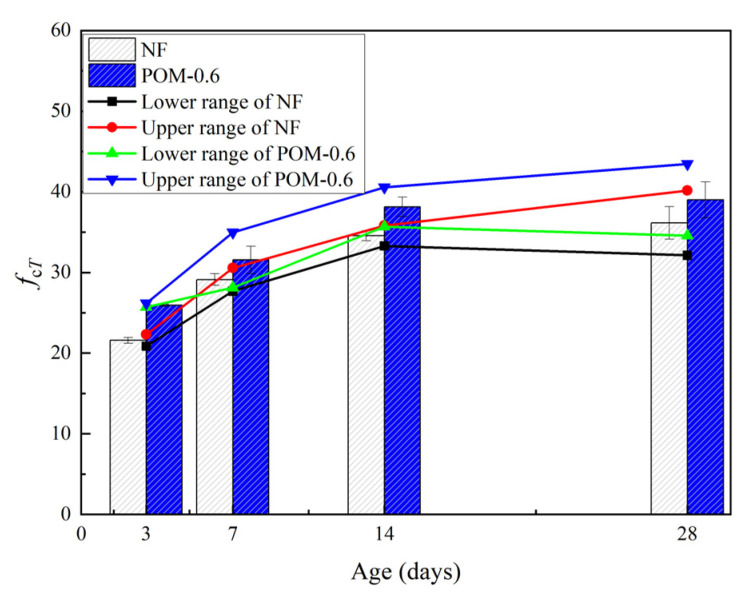
Test results of *f_cT_* for NF and POM0.6 SWSSC specimens with different concrete ages.

**Figure 10:**
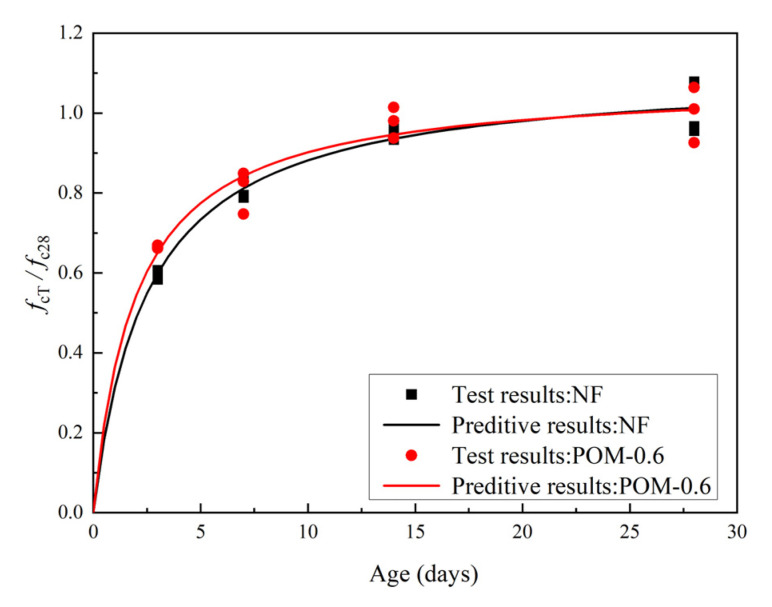
Relations between *f_cT_*/*f_c28_* and concrete age.

**Figure 11:**
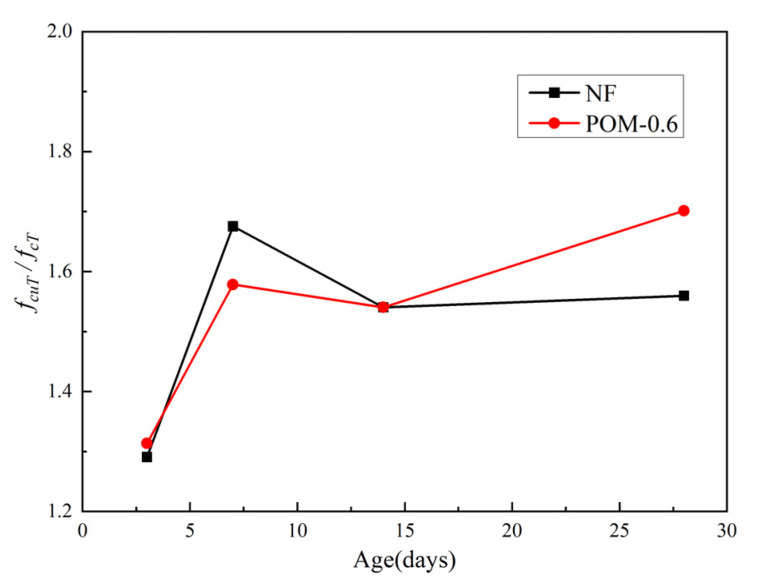
Relations between *f_cuT_*/*f_cT_* and concrete age.

The axial compressive test results of NF and POM0.6 SWSSC specimens with different ages are shown in Table 6. For the axial compressive strength (*f_cT_*) of the test specimens, the standard deviation ranged from 0.12 to 2.22, and the COV ranged from 0.0045 to 0.0568. To quantify *f_cT_* of NF and POM0.6 SWSSC specimens with different ages, the mean value of three specimens was calculated, as shown in Table 6 and Figure 9. *f_cT_* of NF and POM0.6 SWSSC specimens were shown to be positively related to concrete age. Considering the uncertainty of test results, the lower and upper ranges of 95% confidence interval for NF and POM0.6 SWSSC specimens with different ages were introduced, as shown in Table 6 and Figure 9. The test results showed that the usage of POM fibers enhanced the *f_cT_* of NF and POM0.6 SWSSC specimens with different ages. Lower and upper ranges of POM0.6 SWSSC specimens with different ages were larger than those of NF SWSSC specimens with different ages, respectively. Similar to the analysis conducted in Section 6.1, the dimensionless processing was performed to investigate the *f_cT_* of NF and POM0.6 SWSSC specimens with different ages objectively, as shown in Figure 6. Based on the test results of *f_cT_*/*f_c28_*, *f_c_* of NF SWSSC specimens followed the similar trend to that of POM0.6 SWSSC specimens. To predict the *f_cT_* of NF and POM0.6 SWSSC accurately, the predictive equations were also proposed. Based on the axial compressive test results, the equations for axial compression strength of SWSSC with different ages were proposed, as shown in Equations (8) and (9), where R^2^ = 0.97 and 0.90 for NF and POM0.6 SWSSC, respectively. The comparison between test results and the predicted results from Equations (8) and (9) indicated that the proposed equations could be used to predict the *f_cT_* of NF and POM0.6 SWSSC with different ages accurately, as shown in Figure 10. Relations between *f_cuT_* and *f_cT_* changed with increases in the concrete age, as shown in Figure 11. The ratios *f_cuT_*/*f_cT_* of NF and POM0.6 SWSSCs followed the similar trend. When the concrete age increased from 14 d to 28 d, the increase in *f_cuT_*/*f_cT_* of NF SWSSC was smaller than that of POM0.6 SWSSC.

**Figure 12:**
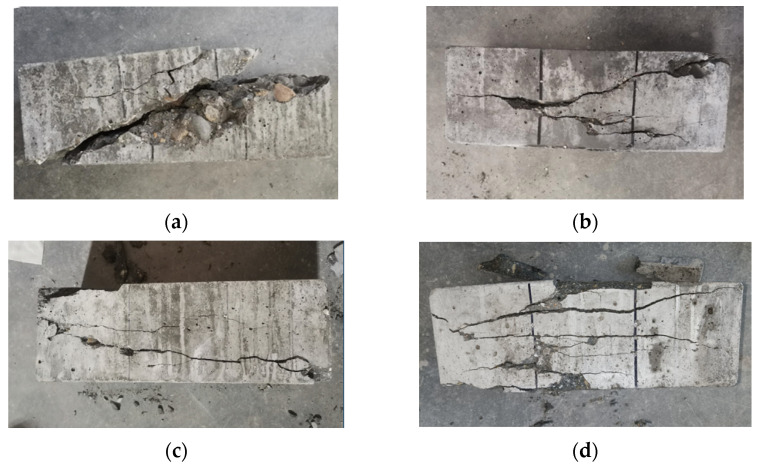
Failure performance of NF specimens with different ages after axial compressive test: (**a**) 3 d; (**b**) 7 d; (**c**) 14 d; and (**d**) 28 d.

**Figure 13:**
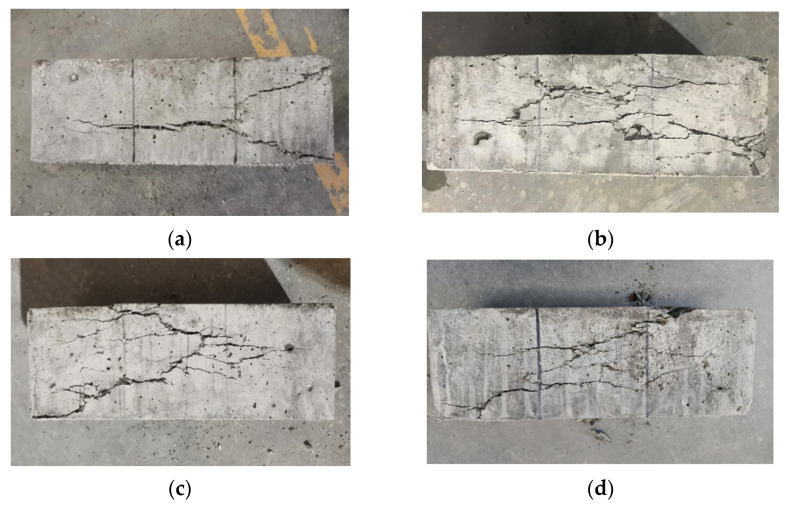
Failure performance of POM0.6 specimens with different ages after axial compressive test: (**a**) 3 d; (**b**) 7 d; (**c**) 14 d; and (**d**) 28 d.

After axial compressive test, failure performances of NF and POM0.3 SWSSC specimens with different ages were reported, as shown in Figure 12 and Figure 13. For NF specimens, vertical microcracks were observed at the beginning of loading. With increases in the load, microcracks were merged into major cracks. It was obvious that cracks penetrated the NF specimens, especially for the NF specimens with 7 d. Cracks in NF specimens with 7 d developed in the cement but did not pass through the coarse aggregate. With increasing concrete age, both coarse aggregate and cement cracked, as shown in Figure 12. Concrete spalling was observed during the axial compressive test of NF specimens. As for POM0.6 specimens, there were connections between concrete on both sides of cracks, which was provided by the POM fiber. The stress concentration in the concrete was alleviated by the POM fiber, which delayed the development of microcracks. Furthermore, no clear separation of concrete specimens was observed. The integrity of test specimens almost remained because of the bridging effect caused by POM fibers, which was also observed in [33].
NF  $f_{cT}=\left(\frac{T}{2.288+0.906T}\right)f_{c28}$
  R^2^ = 0.96  (8)POM0.6  $f_{cT}=\left(\frac{T}{1.821+0.927T}\right)f_{c28}$
  R^2^ = 0.89  (9)

### 5.3. Splitting Tensile Property

**Figure 14:**
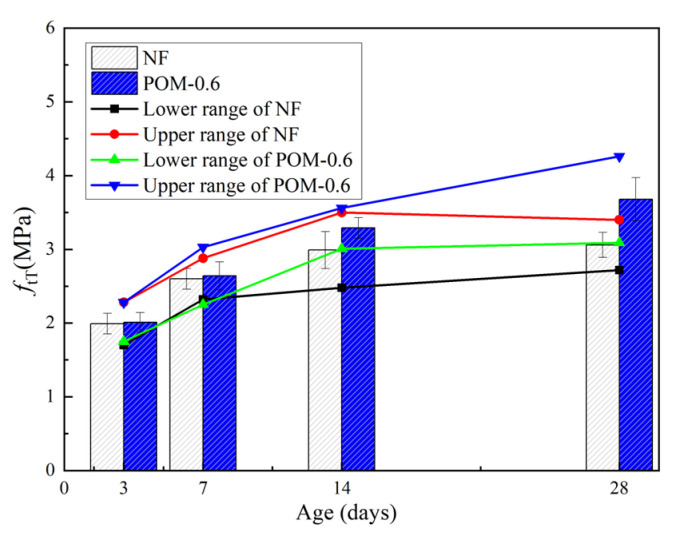
Test results of *f_tT_* for NF and POM0.6 SWSSC specimens with different concrete ages.

**Figure 15:**
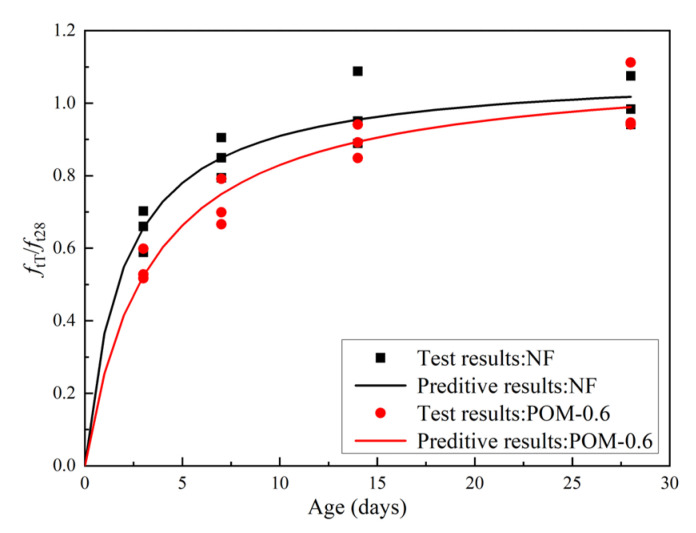
Relations between *f_tT_*/*f_t28_* and concrete age.

**Figure 16:**
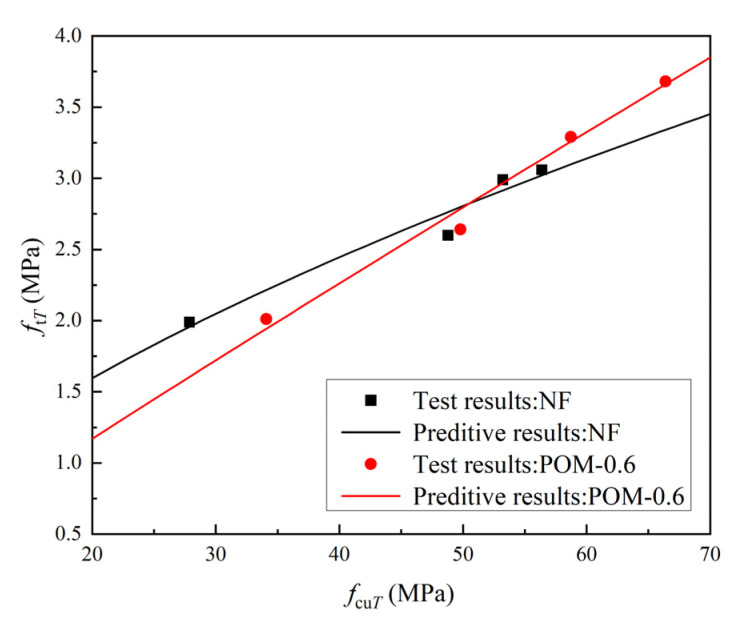
Relations between *f*_t*T*_ and *f*_cu*T*_.

Table 7 presents the mechanical properties of NF and POM0.6 SWSSC specimens with different ages after the splitting tensile test. The standard deviation of splitting tensile strength (*f_tT_*) ranged from 0.13 to 0.29, and the COV ranged from 0.0423 to 0.0853. The mean value of three test specimens was taken to quantify *f_tT_* of NF and POM0.6 SWSSC specimens with different ages, as shown in Table 7 and Figure 14. *f_tT_* of NF and POM0.6 SWSSC specimens shows an increase with the rising concrete age. When the strength of cement was low, enhancing effect of POM fibers on *f_tT_* of SWSSC specimens was not that obvious. Hence, when the concrete age was small (3 d and 7 d), differences in *f_tT_* between NF and POM0.6 SWSSC specimens might be ignored, as shown in Figure 14. When the concrete age was relatively large (14 d and 28 d), POM0.6 SWSSC specimens had better splitting tensile properties, which gradually increased. *f_tT_* increased greatly in the first 7 days and then increased slowly. On the seventh day, the *f_tT_* of NF and POM0.6 SWSSC increased to 85.0% and 71.7% of the *f_t_*, respectively. The lower and upper ranges of 95% confidence interval for NF and POM0.6 SWSSC specimens with different ages are introduced in Table 7 and Figure 14. The relations between *f_tT_*/*f_t28_* and concrete age is shown in Figure 15, showing that *f_tT_* of NF SWSSC specimens developed faster than that of POM0.6 SWSSC specimens, even though they followed the similar trend. Equations (10) and (11) are proposed to predict the *f_tT_* of NF and POM0.6 SWSSC accurately, with R^2^ = 0.84 and 0.91, respectively. In Figure 15, the comparison indicated that the proposed equations could be used to predict the *f_tT_* of NF and POM0.6 SWSSC with different ages accurately. Furthermore, relations between *f_tT_* and *f_cuT_* were investigated, and the predictive equations were proposed. The comparison between test results and results from Equations (12) and (13) indicate that the proposed equations could be used to predict the relations between *f_tT_* and *f_cuT_* accurately, as shown in Figure 16. 

**Figure 17:**
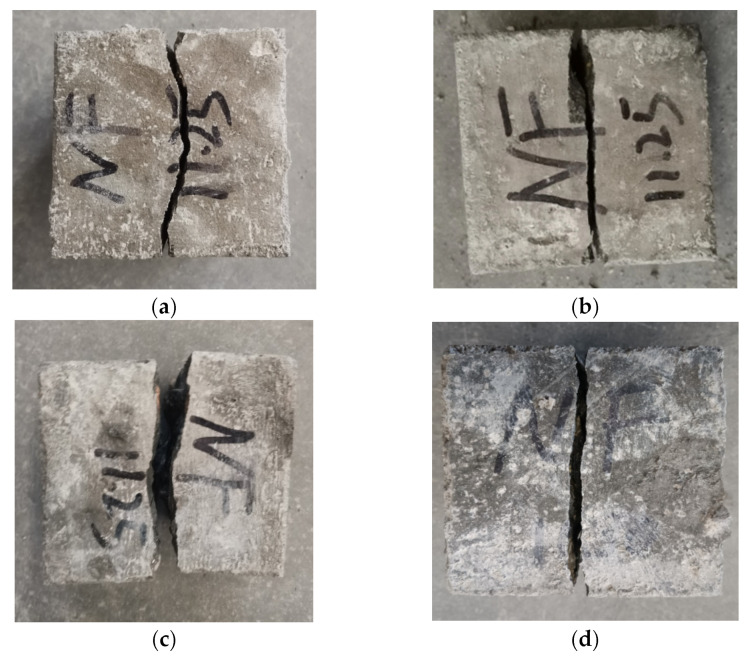
Failure performance of NF specimens with different ages after splitting tensile test: (**a**) 3 d; (**b**) 7 d; (**c**) 14 d; and (**d**) 28 d.

**Figure 18:**
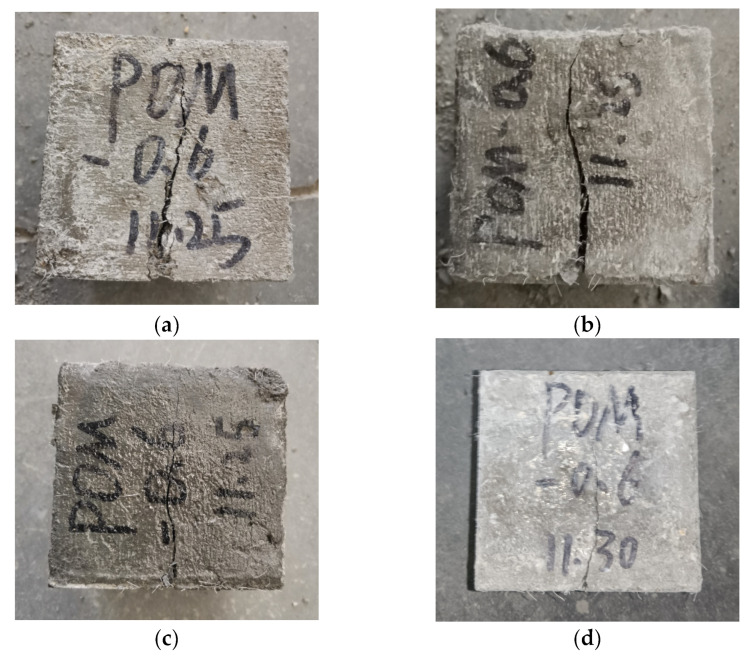
Failure performance of POM0.6 specimens with different ages after splitting tensile test: (**a**) 3 d; (**b**) 7 d; (**c**) 14 d; and (**d**) 28 d.

The failure performances of NF and POM0.6 SWSSC specimens with different concrete ages were analyzed based on the results of the splitting tensile test. For NF SWSSC specimens, the crack occurred in the middle of specimens, as shown in Figure 17. Different from the NF SWSSC specimens, the POM0.6 SWSSC specimens can remain intact after failure, instead of being decomposed into two parts (Figure 18). With increases in concrete age, strength of cement increased correspondingly. The POM fiber was firmly wrapped in concrete, and the development of crack was restrained by the POM fibers, which changed the failure mode of POM0.6 SWSSC specimens. With increases in concrete age, the width of the crack decreased correspondingly.
NF  $f_{tT}=\left(\frac{T}{1.818+0.918T}\right)f_{t28}$
  R^2^ = 0.84  (10)POM0.6  $f_{tT}=\left(\frac{T}{0.903T+3.024}\right)f_{t28}$
  R^2^ = 0.91  (11)NF  $f_{tT}=0.252\times f_{\mathrm{cu}T}{}^{0.616}$
  R^2^ = 0.95  (12)POM0.6  $f_{tT}=0.068\times f_{\mathrm{cu}T}{}^{0.950}$
  R^2^ = 0.98  (13)

### 5.4. Flexural Property

**Figure 19:**
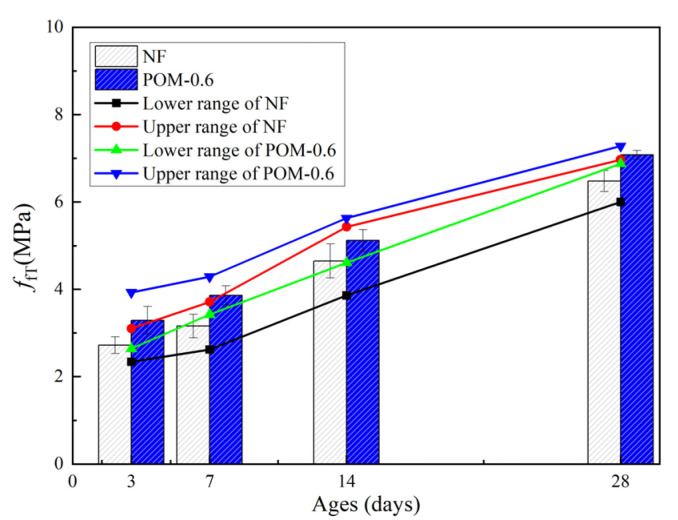
Test results of *f*_fT_ for NF and POM0.6 SWSSC specimens with different concrete ages.

**Figure 20:**
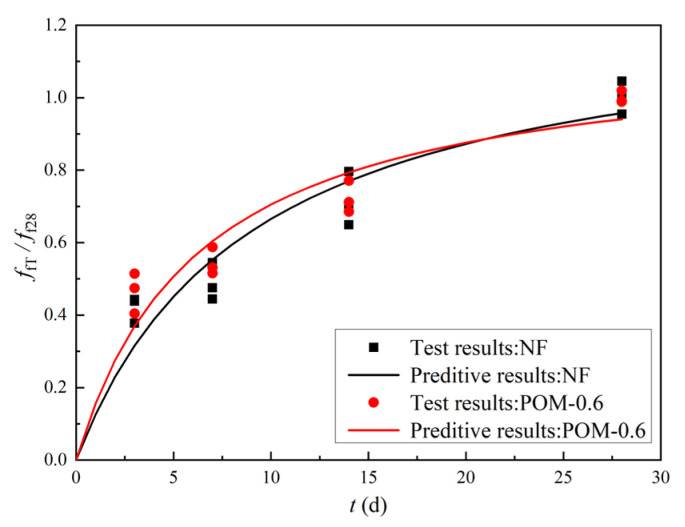
Relations between *f_fT_*/*f_f28_* and concrete age.

**Figure 21:**
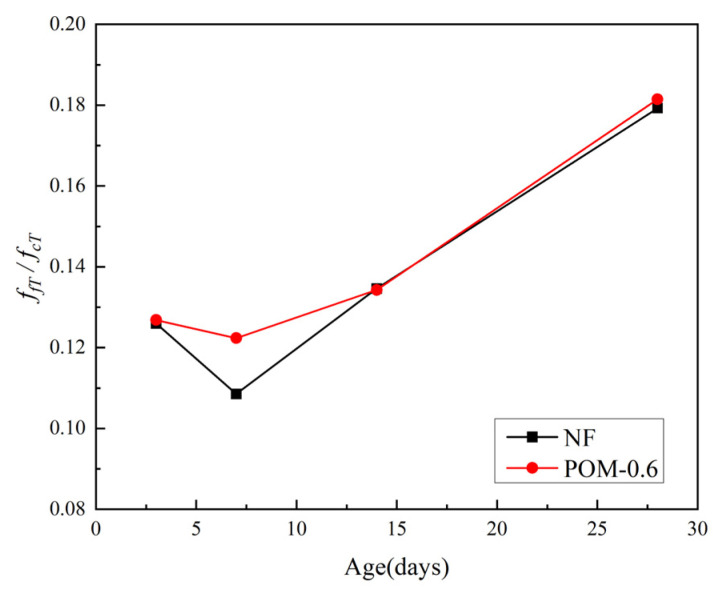
Relations between *f_fT_* and *f_cT_*.

The test results of flexural strength (*f_fT_*) of NF and POM0.6 SWSSC specimens with different ages were obtained after the mechanical test, as shown in Table 8, where the standard deviation and the COV are listed. For all the test specimens, the standard deviation of flexural strength ranged from 0.10 to 0.39, and the COV ranged from 0.0140 to 0.0982. The mean value of three test specimens was calculated to quantify *f_fT_* of NF and POM0.6 SWSSC specimens with different ages, as shown in Figure 19. The lower and upper ranges of 95% confidence interval for NF and POM0.6 SWSSC specimens with different ages are introduced in Table 8 and Figure 19. The test results indicated that the increases in concrete age led to the increase in *f_fT_* of NF and POM0.6 SWSSC specimens. As shown in Figure 20, the *f_fT_*/*f_f28_* ratios of NF and POM0.6 SWSSC specimens followed a similar trend. The *f_fT_*/*f_f28_* ratio of POM0.6 SWSSC specimens was larger than that of NF SWSSC specimen when the concrete age was relatively small (3 d and 7 d). With increases in concrete age, the differences in *f_fT_*/*f_f28_* between NF and POM0.6 SWSSC specimens decreased gradually. Different from the *f_cuT_, f_fT_* of NF and POM0.6 SWSSC specimens also increased significantly after the seventh day. The equations for the flexural strength of NF and POM0.6 SWSSC were proposed based on test results, as presented in Equations (14) and (15), where R^2^ = 0.96 and 0.97, respectively. The comparison indicated that Equations (14) and (15) can be used to predict the *f_fT_* of NF and POM0.6 SWSSC with different ages accurately, as shown in Figure 20. Furthermore, to predict the *f_fT_* of concrete effectively, the relation between *f_fT_* and *f_cT_* was important. For NF and POM0.6 SWSSC in this study, the relations between *f_fT_* and *f_cT_* were quantified based on test results, as shown in Figure 21. For NF and POM0.6 SWSSC specimens, *f_fT_*/*f_cT_* ratio decreased in the early stage, and reached the minimum value on the seventh day. Next, with increases in concrete age, the *f_fT_*/*f_cT_* increased, correspondingly. This phenomenon showed that the increase rate of *f_fT_* in the early stage was slower than that of *f_cT_*, and POM fibers could play a beneficial role in improving *f_fT_* in the early stage.

**Figure 22:**
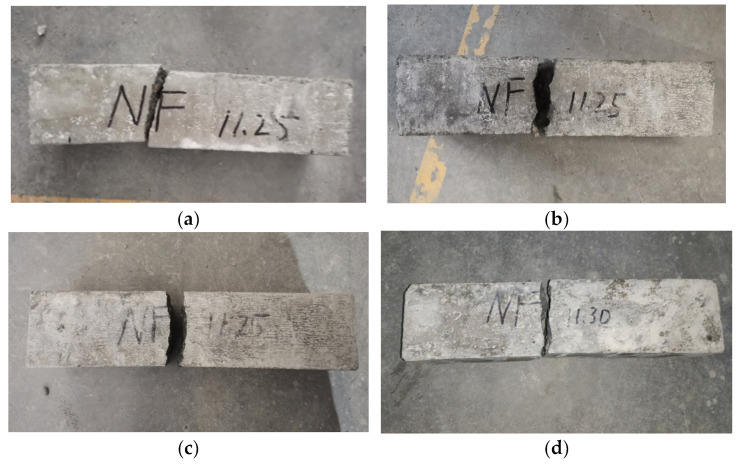
Failure performance of flexural test for NF SWSSC specimens with different ages: (**a**) 3 d; (**b**) 7 d; (**c**) 14 d; (**d**) 28 d.

**Figure 23:**
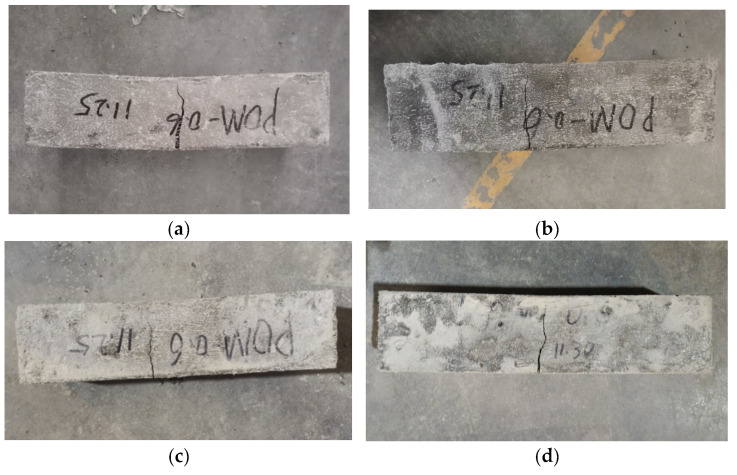
Failure performance of flexural test for POM0.6 SWSSC specimens with different ages: (**a**) 3 d; (**b**) 7 d; (**c**) 14 d; and (**d**) 28 d.

The failure performances of NF and POM0.6 SWSSC specimens with different ages were discussed, based on test results, as shown in Figure 22 and Figure 23. Without the POM fiber, no bridging effect was observed in the concrete, where the clear crack divided the NF SWSSC test specimens into two parts, and no connection was observed. The brittle failure mode was defined for NF SWSSC specimens. For POM0.6 SWSSC specimens, even though there was a clear crack occurring in the test specimens, the connection between the concretes was observed, and the bridging effect was noticed. The development of crack was shown to restrain the POM fiber effectively, and the integrity of POM0.6 SWSSC specimens almost remained after the flexural test. The above features of flexural test specimens were similar to those of splitting tensile test specimens. Because of the POM fiber, flexural test specimens with different ages performed the ductile failure mode.
NF  $f_{fT}=\left(\frac{T}{7.133+0.790T}\right)f_{f28}$
  R^2^ = 0.97  (14)POM0.6  $f_{fT}=\left(\frac{T}{5.529+0.866T}\right)f_{f28}$
  R^2^ = 0.97  (15)

## 6. Discussion

### 6.1. Stress–Strain Curve of SWSSC 

**Figure 24:**
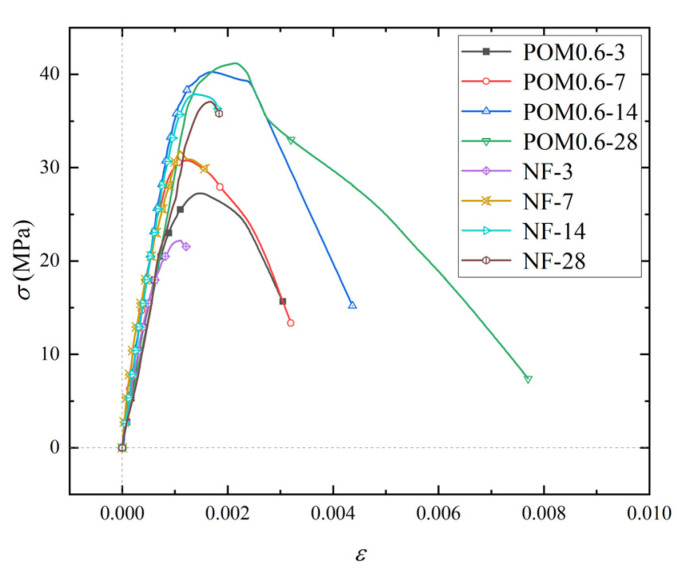
Stress–strain curve of SWSSC specimens with different ages.

**Figure 25:**
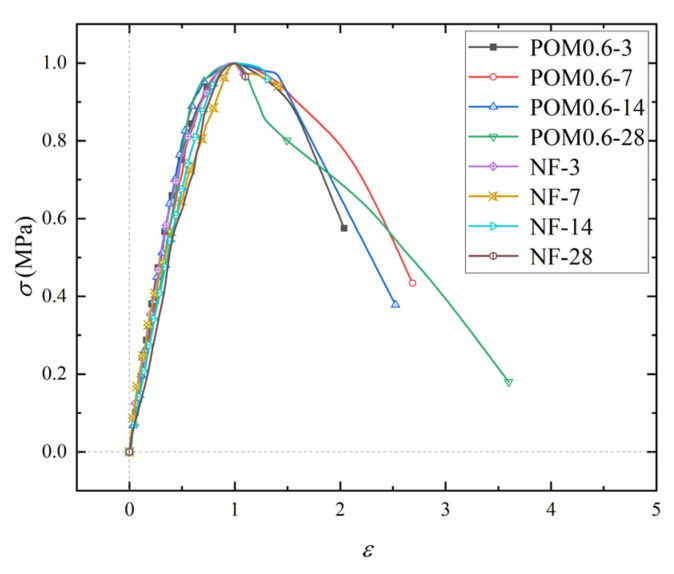
Dimensionless stress–strain curve of SWSSC specimens with different ages.

The maximum values of various mechanical properties for SWSSC specimens were introduced in Section 5. In order to further clarify the change process of stress and for SWSSC specimens during compression, the stress–strain curves of NF and POM0.6 SWSSC specimens were obtained based on the axial compressive test, as shown in Figure 24. In order to better observe the morphology of the stress–strain curve, the stress–strain data were dimensionless (Figure 25). The stress–strain curve of NF and POM0.6 SWSSC could be divided into four stages. When the stress was less than 0.3*f*_c_, the stress–strain curve of NF and POM0.6 SWSSC was approximately linear, indicating that the deformation of NF and POM0.6 SWSSC was elastic at this stage. When 0.3*f*_c_ < *σ* < 0.85*f*_c_, the slope of stress–strain curves decreased slightly. Microcracks in concrete were produced from the loading end and gradually extend to the interior of the specimens at this stage. Then, with the increase in stress (0.85*f*_c_ < *σ* < *f*_c_), macrocracks began to appear on the surface of the specimens and developed rapidly due to the transverse deformation. The POM fibers passing through the cracks prevented the concrete from cracking, which reduced the rate of crack propagation. Therefore, the curve slope of POM0.6 SWSSC specimens at this stage was smaller than that of NF SWSSC specimens. When the stress reached *f*_c_, the cracks inside the specimen penetrated and formed a failure surface. Macroscopic cracks appeared on the surface of the SWSSC specimens; then the specimens were broken. For POM0.6 SWSSC, the fibers passing through the failure surface were broken or pulled from the concrete matrix, which is shown in Figure 13. Due to the brittle failure characteristics of NF SWSSC, the NF SWSSC specimen was broken immediately after the stress reached *f*_c_. The extensometers could not be fixed on the specimens effectively, resulting in an inability to obtain the data of the failure stage. Due to the good anti-cracking effect of POM fibers, the fiber passing through the crack and near the crack tip would transfer the stress to the upper and lower surfaces of the crack, which reduced the stress concentration at the crack tip and slowed the failure process. Therefore, the complete stress–strain curve of POM0.6 SWSSC specimens under uniaxial compression could be obtained. It further illustrated that POM fibers have a significant effect on improving the ductility of concrete. 

**Figure 26:**
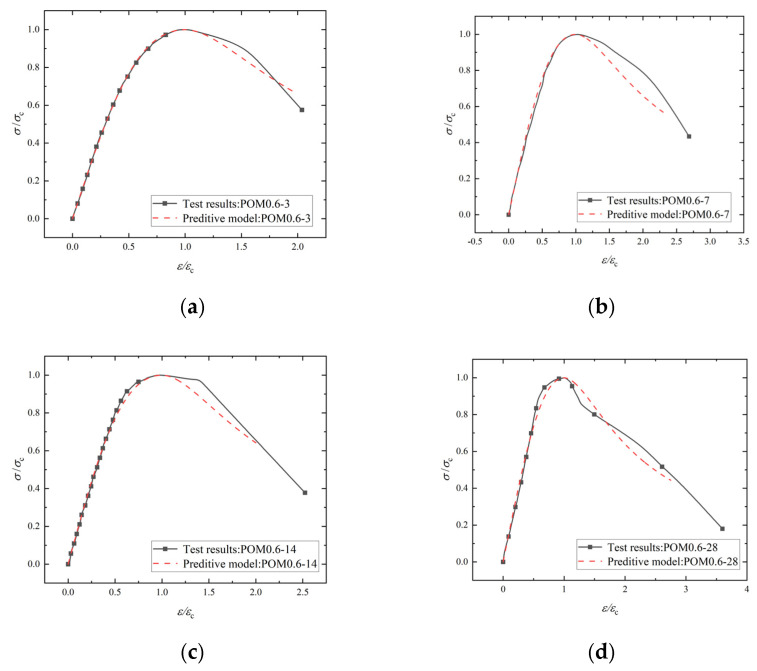
Fitting results of stress–strain curve of POM SWSSC: (**a**) POM0.6-3; (**b**) POM0.6-7; (**c**) POM0.6-14; and (**d**) POM0.6-28.

**Figure 27:**
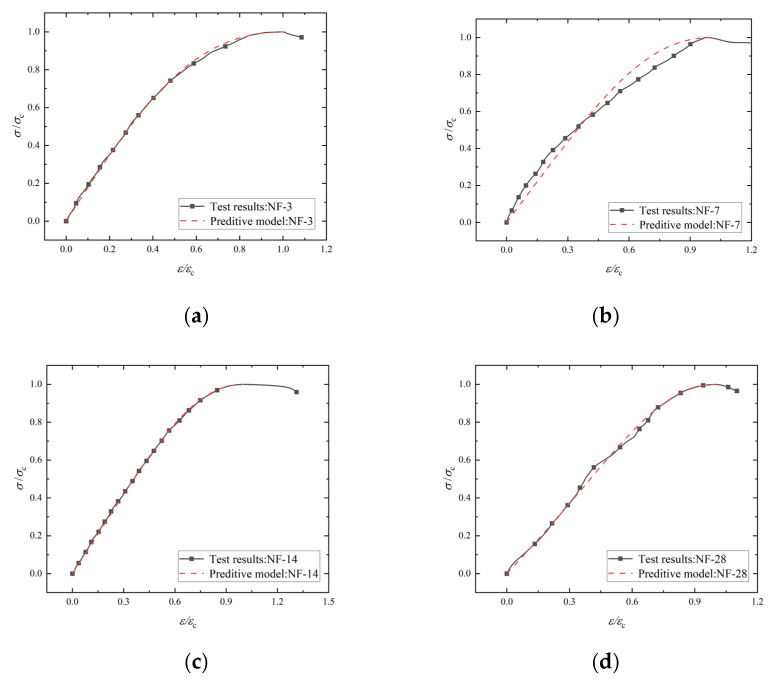
Fitting results of stress–strain curve of NF SWSSC: (**a**) NF-3; (**b**) NF-7; (**c**) NF-14; and (**d**) NF-28.

**Figure 28:**
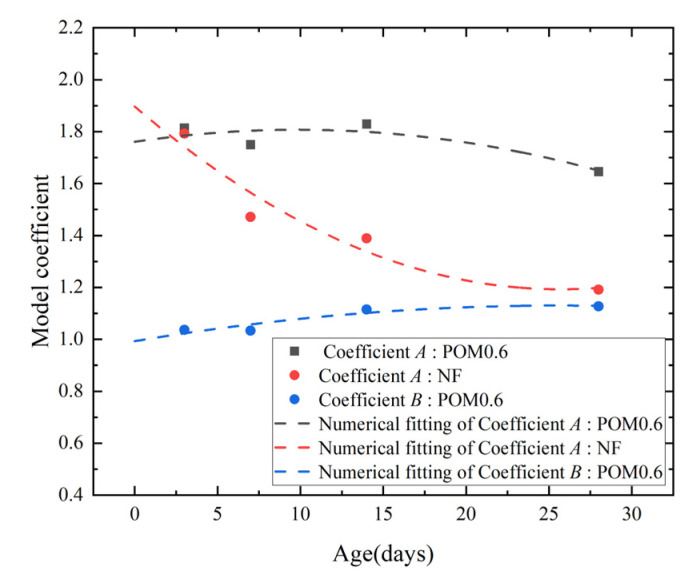
Model coefficients of POM and NF SWSSC with different ages.

In order to describe the stress–strain curve characteristics of NF and POM0.6 SWSSC quantitatively, a numerical model was introduced, as shown in Equations (16)–(18). It should be noted that the model of NF SWSSC was only used for the stage before *f*_c_ because the data of the failure stage had not been obtained. The comparison between the model and test results is shown in Figure 26 and Figure 27. The model can be found to simulate the experimental results well. Figure 28 represents the variation law of model coefficients *A* and *B* with concrete age. The coefficient *A* of NF SWSSC decreased gradually with the increase in concrete age. The model coefficient *A* of POM0.6 SWSSC was larger than that of NF SWSSC and changed little with the increase in concrete age. The model coefficient *B* of POM0.6 specimen also changed slightly with the increase in concrete age, which illustrated that the POM fiber made the shape of the stress–strain curve of SWSSC at early concrete age more stable. The model coefficients *A* and *B* were fitted, as shown in Equations (19)–(21). The stress–strain curve of NF and POM0.6 SWSSC at any concrete age could be predicted, as shown in Equations (16)–(21).

  $y=\left\{\begin{array}{c} \frac{Ax}{1+\left(2A-3\right)x^{2}+\left(2-A\right)x^{3}},0\leq x\leq 1\\ \frac{x}{B{\left(x-1\right)}^{2}+x},x\geq 1 \end{array}\right.$
  (16)
  $x=\varepsilon /\varepsilon_{c}$
  (17)
  $y=\sigma /f_{c}$
  (18)POM0.6  $A=-4.79\times 10^{-4}T^{2}+9.45\times 10^{-3}T+1.76$
  (19)NF  $B=-2.09\times 10^{-4}T^{2}+1.07\times 10^{-2}T+0.99$
  (20)POM0.6  $A=1.07\times 10^{-3}T^{2}-5.49\times 10^{-2}T+1.90$
  (21)

### 6.2. Microstructure

**Figure 29:**
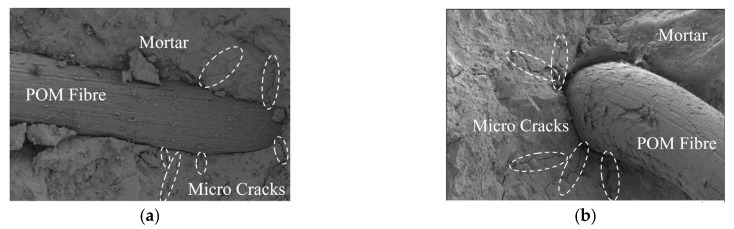
SEM results of POM0.6 SWSSC: (**a**) Along the fracture surface; (**b**) perpendicular to the fracture surface.

The mechanical properties of fiber-reinforced concrete were determined by its microstructure, which was closely related to the bond performance between the fibers and mortar. In this study, the Gemini SEM 300 field emission scanning electron microscope was used to observe the microstructure of POM SWSSC, and the reinforcing mechanism of the POM fiber was discussed. The POM SWSSC specimen was immersed in absolute ethanol solution for 24 h to ensure that there was no water in the specimen. After that, the specimen was coated to improve the conductivity, and its microstructure was observed. The interface between the POM fibers and concrete matrix is shown in Figure 29. The POM fiber was shown to be closely combined with the mortar. A few radioactive microcracks exist at the interface between the POM fiber and mortar, which indicated that the force could be effectively transferred between the POM fiber and mortar. Therefore, when the microcracks in the concrete propagated, the POM fiber located at the microcracks could be effectively stressed, which hindered the propagation of microcracks to a certain extent. Based on the discussion in Section 5.2, when the POM SWSSC was broken, the POM fiber bridged the cracks, which not only reduced the crack width but also ensured the integrity of the concrete after being broken. POM fibers crisscrossed in the concrete consumed energy together, which delayed the propagation of cracks and significantly improved the plastic deformation capacity of the SWSSC. When the SWSSC was in tension, the reaction force was transmitted to the fibers through the bonding force [36,37]. Because the POM fiber has outstanding tensile strength compared with the mortar, splitting tensile properties and flexural properties of the concrete can be improved significantly. 

## 7. Conclusions

In the study, effects of POM fibers on mechanical properties of SWSSC with different concrete ages were investigated experimentally. A total of 96 test specimens and 2 different concrete mixtures were included in the experiments. The main contributions of this study included the following: 

The usage of POM fibers reduced workability of SWSSC. Compared with those of NF mixture, the slump and expansibility of the POM0.6 mixture decreased by 25.7% and 20.7%, respectively. The POM fibers had an enhancing effect on *f_cuT_*. The *f_cuT_* of NF SWSSC specimens developed faster than that of POM0.6 SWSSC specimens at early age. The Equations (6) and (7) are proposed to quantify *f_cuT_* of NF and POM0.6 SWSSC with different ages accurately. 

With increases in age, *f_cT_* of NF and POM0.6 SWSSC specimens increased. Because of the enhancing effect of POM fibers, *f_cT_* of POM0.6 SWSSC specimens was larger than that of NF SWSSC specimens. *f_cT_* of NF SWSSC specimens followed the similar trend to that of POM0.6 SWSSC specimens. The Equations (8) and (9) are proposed to quantify *f_cT_* of NF and POM0.6 SWSSC with different ages accurately. 

When the concrete age was relatively small (3 d and 7 d), differences in *f_tT_* between NF and POM0.6 SWSSC specimens might be ignored. With the increases in concrete age, POM0.6 SWSSC specimens performed better splitting tensile properties. Equations (10)–(13) are proposed to predict the *f_tT_* and *f_tT_*/*f_cT_* of NF and POM0.6 SWSSC. 

*f_fT_* of NF and POM0.6 SWSSC specimens was positively related with the concrete age. With increases in concrete age, *f_fT_* of NF and POM0.6 SWSSC specimens followed the similar trend. Equations (14)–(17) are proposed to predict the *f_fT_* and *f_fT_*/*f_cT_* of NF and POM0.6 SWSSC. 

POM fibers made the shape of stress–strain curve of SWSSC at early age more stable. A numerical model was introduced, which can predict the stress–strain curve of NF and POM0.6 SWSSC at any age. SEM results show that the force can be effectively transferred between the POM fiber and cement matrix. POM fibers could significantly improve the splitting tensile properties and flexural properties of the concrete.

## Figures and Tables

**Table 1:** Comparison among different fibers [29,34].

Type	Tensile Strength (MPa)	Elongation (%)	Melting Point (°C)	Elastic Modulus (GPa)	Density (kg/m^3^)
POM	967	18	165	8	1400
Steel	200~2760	0.5~35	1370	200	7800
Glass	1034~3792	1.5~3.5	860	72	2500~2700
Carbon	1550~6960	2.5~3.2	3000	159~965	1800
Basalt	3200	3.1	1200	89	2700
PP	400	30	180	3.85	910

**Table 2:** Comparison in chemical composition among the fresh water, seawater, and sea–sand.

	Cl^−^	SO_4_^2−^	Na^+^	K^+^	Mg^2+^	Ca^2+^	Total
Fresh water (mg/L)	12.3	36.8	8.7	2.8	9.6	53.1	123.3
Seawater (mg/L)	19,365.5	2537.5	11,208.7	389.9	1321.7	395.8	35,219.1
Sea–sand (mg/kg)	7.4	34.9	14.2	4.0	3.0	13.8	77.3

**Table 3:** Proportions of NF and POM0.6 mixtures.

Types	Cement (kg/m^3^)	Fly Ash (kg/m^3^)	Mineral Powder (kg/m^3^)	Sea–Sand (kg/m^3^)	Coarse Aggregate (kg/m^3^)	Seawater (kg/m^3^)	POM Fiber (kg/m^3^)	W/C Ratio
NF	264	88	88	831	1016	160	0	0.36
POM0.6							8.4	

**Table 4:** Workability of different types of SWSSC.

Types	Slump (mm)	Expansibility 1 (mm)	Expansibility 2 (mm)	Expansibility (mm)
NF	175	291	286	290
POM0.6	130	235	222	230
C20 SSRCA [26]	60	-	-	-
C30 SSRCA [26]	35	-	-	-
C40 SSRCA [26]	210	-	-	-
C50 SSRCA [26]	200	-	-	-

**Table 5:** Cube compressive strength of NF and POM0.6 SWSSC specimens with different ages.

Number	*f_cuT_* (MPa)	*f_cuT-mean_* (MPa)	Standard Deviation	COV	95% Confidence Interval	
					Lower Range	Upper Range
NF-3-1	29.16					
NF-3-2	27.74	27.87	1.00	0.0359	25.87	29.87
NF-3-3	26.72					
POM0.6-3-1	35.25					
POM0.6-3-2	36.93	34.08	2.93	0.0859	28.22	39.93
POM0.6-3-3	30.05					
NF-7-1	48.45					
NF-7-2	48.13	48.79	0.72	0.0148	47.35	50.24
NF-7-3	49.80					
POM0.6-7-1	52.03					
POM0.6-7-2	48.45	49.79	1.59	0.0320	46.61	52.98
POM0.6-3-3	48.9					
NF-14-1	54.62					
NF-14-2	54.27	53.22	1.74	0.0328	49.73	56.70
NF-14-3	50.76					
POM0.6-14-1	56.69					
POM0.6-14-2	60.87	58.73	1.71	0.0291	55.31	62.15
POM0.6-14-3	58.63					
NF-28-1	58.67					
NF-28-2	58.15	56.38	2.87	0.0510	50.64	62.13
NF-28-3	52.33					
POM0.6-28-1	65.29					
POM0.6-28-2	66.36	66.39	0.91	0.0138	64.56	68.22
POM0.6-28-3	67.53					

**Table 6:** Axial compressive strength of NF and POM0.6 specimens with different ages.

Number	*f_cT_* (MPa)	*f_cT-mean_* (MPa)	Standard Deviation	COV	95% Confidence Interval	
					Lower Range	Upper Range
NF-3-1	21.09					
NF-3-2	21.94	21.59	0.36	0.0169	20.86	22.32
NF-3-3	21.75					
POM0.6-3-1	25.91					
POM0.6-3-2	25.82	25.94	0.12	0.0045	25.71	26.18
POM0.6-3-3	26.10					
NF-7-1	30.13					
NF-7-2	28.50	29.12	0.72	0.0247	27.69	30.56
NF-7-3	28.74					
POM0.6-7-1	33.14					
POM0.6-7-2	29.17	31.55	1.71	0.0543	28.12	34.97
POM0.6-3-3	32.33					
NF-14-1	33.74					
NF-14-2	34.63	34.55	0.63	0.0183	33.29	35.81
NF-14-3	35.28					
POM0.6-14-1	36.58					
POM0.6-14-2	39.56	38.13	1.22	0.0320	35.69	40.57
POM0.6-14-3	38.25					
NF-28-1	34.94					
NF-28-2	34.53	36.15	2.01	0.0555	32.13	40.17
NF-28-3	38.98					
POM0.6-28-1	39.41					
POM0.6-28-2	41.52	39.02	2.22	0.0568	34.58	43.46
POM0.6-28-3	36.13					

**Table 7:** Splitting tensile strength of NF and POM0.6 specimens with different ages.

Number	*f_tT_* (MPa)	*f_tT-mean_* (MPa)	Standard Deviation	COV	95% Confidence Interval	
					Lower Range	Upper Range
NF-3-1	2.60					
NF-3-2	2.77	1.99	0.14	0.0726	1.70	2.28
NF-3-3	2.43					
POM0.6-3-1	2.45					
POM0.6-3-2	2.57	2.01	0.13	0.0661	1.75	2.28
POM0.6-3-3	2.91					
NF-7-1	2.60					
NF-7-2	2.77	2.60	0.14	0.0534	2.32	2.88
NF-7-3	2.43					
POM0.6-7-1	2.45					
POM0.6-7-2	2.57	2.64	0.19	0.0737	2.25	3.03
POM0.6-3-3	2.91					
NF-14-1	2.91					
NF-14-2	2.72	2.99	0.25	0.0853	2.48	3.50
NF-14-3	3.33					
POM0.6-14-1	4.09					
POM0.6-14-2	3.48	3.29	0.14	0.0423	3.01	3.56
POM0.6-14-3	3.46					
NF-28-1	3.01					
NF-28-2	3.29	3.06	0.17	0.0559	2.72	3.40
NF-28-3	2.88					
POM0.6-28-1	4.09					
POM0.6-28-2	3.48	3.68	0.29	0.0795	3.09	4.26
POM0.6-28-3	3.46					

**Table 8:** Flexural strength of NF and POM0.6 specimens with different ages.

Number	*f_fT_* (MPa)	*f_fT-mean_* (MPa)	Standard Deviation	COV	95% Confidence Interval	
					Lower Range	Upper Range
NF-3-1	2.84					
NF-3-2	2.87	2.72	0.19	0.0703	2.34	3.10
NF-3-3	2.45					
POM0.6-3-1	3.36					
POM0.6-3-2	3.64	3.29	0.32	0.0982	2.64	3.93
POM0.6-3-3	2.86					
NF-7-1	3.08					
NF-7-2	2.88	3.16	0.27	0.0859	2.62	3.71
NF-7-3	3.53					
POM0.6-7-1	3.65					
POM0.6-7-2	4.16	3.86	0.22	0.0568	3.42	4.29
POM0.6-3-3	3.76					
NF-14-1	5.16					
NF-14-2	4.57	4.65	0.39	0.0843	3.86	5.43
NF-14-3	4.21					
POM0.6-14-1	4.85					
POM0.6-14-2	5.46	5.12	0.25	0.0498	4.61	5.63
POM0.6-14-3	5.04					
NF-28-1	6.78					
NF-28-2	6.19	6.48	0.24	0.0372	6.00	6.97
NF-28-3	6.48					
POM0.6-28-1	7.22					
POM0.6-28-2	7.02	7.08	0.10	0.0140	6.88	7.28
POM0.6-28-3	7.00					

## Data Availability

Not applicable.
